# Quantum dot fluorescent microsphere-based immunochromatographic strip for detecting PRRSV antibodies

**DOI:** 10.1007/s00253-024-13125-2

**Published:** 2024-04-04

**Authors:** Rui Yang, Yi Ru, Huibao Wang, Rongzeng Hao, Yajun Li, Tao Zhang, Haixue Zheng, Yong Zhang, Xingxu Zhao

**Affiliations:** 1 College of Veterinary Medicine, Gansu Agricultural University, Lanzhou, 730070 Gansu China; 2 State Key Laboratory for Animal Disease Control and Prevention, College of Veterinary Medicine, Lanzhou University, Lanzhou Veterinary Research Institute, Chinese Academy of Agricultural Sciences, Lanzhou, 730000 Gansu China; 3 China Agricultural Veterinarian Biology Science and Technology Co. Ltd, Lanzhou, 730046 Gansu China; 4 College of Modern Agricultural Engineering, Gansu Forestry Technological College, Tianshui, 741020 Gansu China

**Keywords:** Porcine reproductive and respiratory syndrome, Quantum dot fluorescent microsphere, Immunochromatography strip, Monoclonal antibody

## Abstract

**Abstract:**

Porcine reproductive and respiratory syndrome (PRRS) is an immunosuppressive disease caused by the porcine reproductive and respiratory syndrome virus (PRRSV). Current vaccine prevention and treatment approaches for PRRS are not adequate, and commercial vaccines do not provide sufficient cross-immune protection. Therefore, establishing a precise, sensitive, simple, and rapid serological diagnostic approach for detecting PRRSV antibodies is crucial. The present study used quantum dot fluorescent microspheres (QDFM) as tracers, covalently linked to the PRRSV N protein, to develop an immunochromatography strip (ICS) for detecting PRRSV antibodies. Monoclonal antibodies against PRRSV nucleocapsid (N) and membrane (M) proteins were both coated on nitrocellulose membranes as control (C) and test (T) lines, respectively. QDFM ICS identified PRRSV antibodies under 10 min with high sensitivity and specificity. The specificity assay revealed no cross-reactivity with the other tested viruses. The sensitivity assay revealed that the minimum detection limit was 1.2 ng/mL when the maximum dilution was 1:2,048, comparable to the sensitivity of enzyme-linked immunosorbent assay (ELISA) kits. Moreover, compared to PRRSV ELISA antibody detection kits, the sensitivity, specificity, and accuracy of QDFM ICS after analyzing 189 clinical samples were 96.7%, 97.9%, and 97.4%, respectively. Notably, the test strips can be stored for up to 6 months at 4 °C and up to 4 months at room temperature (18–25 °C). In conclusion, QDFM ICS offers the advantages of rapid detection time, high specificity and sensitivity, and affordability, indicating its potential for on-site PRRS screening.

**Key points:**

• *QDFM ICS is a novel method for on-site and in-lab detection of PRRSV antibodies*

• *Its sensitivity, specificity, and accuracy are on par with commercial ELISA kits*

• *QDFM ICS rapidly identifies PRRSV, aiding the swine industry address the evolving virus*

**Supplementary Information:**

The online version contains supplementary material available at 10.1007/s00253-024-13125-2.

## Introduction

Porcine reproductive and respiratory syndrome (PRRS), one of the major viral diseases caused by porcine reproductive and respiratory syndrome virus (PRRSV), has negatively affected the swine sector for approximately 36 years (Neumann et al. 2005; Du et al. 2017). The disease causes reproductive challenges in pregnant sows and respiratory disorders in pigs. Since its emergence in the USA in 1987, PRRSV has spread worldwide (Elazhary et al. 1991; Wensvoort et al. 1991; Albina 1997), resulting in significant economic losses, especially in countries with intensive pig production (Neumann et al. 2005; Snijder et al. 2013; Dokland 2010).

As a single-stranded positive-sense RNA virus with an envelope of 50–65 nm, PRRSV belongs to the *Arteriviridae* family, genus *Porartevirus* (Dokland 2010). Its genome is approximately 15 kb, with 11 open reading frames (ORFs) encoding 14 non-structural and eight structural proteins (Thiel et al. 1993; Meulenberg et al. 1993; Zhou and Yang 2010; Han et al. 2006; Tian et al. 2007). The membrane (M) protein encoded by *ORF6* and the nucleocapsid (N) protein encoded by *ORF7* are conserved, serving as useful molecular markers for epidemiological investigation (Lopez and Osorio 2004; Lopez et al. 2007; Snijder et al. 2013; Trible et al. 2015; Li et al. 2022a, b). PRRSV can be classified into two genotypes, European (PPRSV-1) and North American (PRRSV-2), with a nucleic acid homology of 60% (Adams et al. 2017). The PRRSV-2 genotype was first reported in 1996 in China. Currently, most epidemic PRRSV strains in China belong to PRRSV-2, including classical PRRSV (C-PRRSV), highly pathogenic PRRSV (HP-PRRSV), NADC30 PRRSV, and NADC34 PRRSV. Owing to the high variability and recombination of PRRSV strains, commercial vaccines do not provide adequate cross-immune protection. Furthermore, there are no effective therapies for PRRSV infections. Therefore, rapid and accurate virus detection is essential for PRRS prevention and control.

Currently, PRRSV detection methods include virus isolation, indirect immunofluorescence assay (IFA), enzyme-linked immunosorbent assay (ELISA), reverse transcription polymerase chain reaction (RT-PCR), and quantitative RT-PCR (qRT-PCR) (Cai et al. 2009; Gibert et al. 2017; Park et al. 2016). However, these methods require strict laboratory conditions, expensive instruments, and skilled technicians, limiting their application in point-of-care testing. Accordingly, there is an urgent need to establish a rapid, simple, and sensitive method for PRRSV detection, particularly in low-resource settings.

Immunochromatographic strips (ICS) are widely used immunoassays with advantages such as rapid detection, low cost, and convenient operation. Although colloidal gold-based ICSs have been used for PRRSV antibody detection, they exhibit low sensitivity (Yu et al. 2018). Recently, quantum dot fluorescent microspheres (QDFM) have gained attention because of their excellent fluorescent properties and potential application as labels for fluorescence immunoassays. QDFM-based ICSs are widely used for detecting viruses (Nguyen et al. 2020), bacteria (Kong et al. 2021; Xu et al. 2013; Li et al. 2020), cancer (Shojaeian et al. 2018; Zhang et al. 2017), and food safety (Singh et al. 2023). Therefore, in the present study, we established antibody detection of PRRSV using QDFM-based ICS, which can monitor the status of PRRSV infection in real-time field conditions.

## Materials and methods

### Materials and reagents

Polyethylene glycol (PEG) (NW1450) was purchased from Merk (Darmstadt, Germany). Carboxyl water-soluble QDFM (Q 2605) was purchased from Wuhan Jiayuan Quantum Dots Co., Ltd. (Wuhan, China). N-(3-dimethylaminopropyl)-N′-ethylcarbodiimide hydrochloride (EDC), N-hydroxysulfosuccinimide sodium salt (NHS), hypoxanthine-aminopterin-thymidine (HAT), and hypoxanthine-thymidine (HT) medium were purchased from Thermo Fisher Scientific (Waltham, MA, USA). The nitrocellulose (NC) membranes (78356403) were purchased from Whatman (Maidstone, UK). The other materials for the test strip, including glass cellulose membranes (JCA0631), water absorption pad (JCA1226), and PVC back plate (J-B6), were purchased from Shanghai Jiening Biotechnology Co. Ltd. (Shanghai, China). An electronic analytical balance model BSA224S-CW was purchased from Sartorius Scientific Instruments (Beijing) Co., LTD. (Beijing, China). The BioDot XYZ distribution platform was purchased by BioDot Corporation (Richmond, CA, USA). An automatic cutter and fluorescent strip reader (JN615) were purchased from Shanghai Jinbiao Biotechnology Co. Ltd. (Shanghai, China). The portable 365-nm UV light was purchased from Jingdong Mall (China). PRRSV antibody test kits (L 351) were purchased from IDEXX (Westbrook, ME, USA).

### Samples and biological materials

PRRSV M and N proteins and N monoclonal antibodies (mAb) were prepared in our laboratory. In addition, foot-and-mouth disease antigen (FMD-O-VP1) and *Senecavirus* A antigen (SVA-VP1) were stored in our laboratory. During 2019–2022, 189 blood samples were collected from three farms in Fujian Province, China, where reproductive problems occasionally occur. Serum samples were obtained and stored at − 20 °C. PRRSV, African swine fever virus (AFSV), FMD-O, *Senecavirus* A (SVA), porcine parvovirus (PP), pseudorabies virus (PR)-positive serum, and healthy pig serum were provided by the State Key Laboratory for Animal Disease Control and Prevention, Lanzhou Veterinary Research Institute, Chinese Academy of Agriculture Sciences.

### Production and identification of M and N proteins

The truncated M protein (NCBI registration number PP378623) and recombinant N protein (NCBI registration number PP378624) were produced according to the M and N genes of the PRRSV CH-1R strain (NCBI registration numbers AY032626: ORF6 and ORF7) in GenBank. The truncated and recombinant gene sequences were sent to Qingke Biological Co., Ltd. (Xi’an, China) for synthesis (Figure S1). Recombinant plasmids pET28a-SUMO-M and pET28a-N were transformed into competent *Escherichia coli* BL21 cells to obtain recombinant *Escherichia coli*. Transformed cells were plated on Luria–Bertani (LB) plates supplemented with 100 mg/L kanamycin. After obtaining single colonies, positive clones were grown in LB broth supplemented with 100 mg/L kanamycin and 1 mmol/L isopropyl-*β*-D-thiogalactopyranoside (IPTG; TakaraBio, Shiga, Japan) at 16 °C with strong shaking for 8 h to induce recombinant protein expression. Cells were harvested and sonicated after the induction period. The truncated M protein and recombinant N protein were purified with a commercial protein purification product (Ni Sepharose HP, GE) and assessed using sodium dodecyl sulfate–polyacrylamide gel electrophoresis (SDS-PAGE) and western blotting using PRRSV-positive serum. The proteins were then divided into 1 mg/mL aliquots-and stored at − 70 °C.

### Production and purification of N monoclonal antibodies

The concentration of the immune antigen (purified PRRSV N protein) was determined, and the volume of the antigen used was calculated. Freund’s adjuvant was added in the same volume and emulsified, then stored for 6–8 h at 4 °C. When stratification did not occur, five BALB/c mice aged 6–8 weeks were immunized using a subcutaneous multi-point injection in the back. The first dose administered was 100 μg, consisting of an equal volume emulsification of PRRSV N and Freund’s complete adjuvant. The second and third booster doses for immunity, given at 14-day intervals, were 50 μg each, consisting of an equal volume emulsification of PRRSV N and Freund’s incomplete adjuvant. On the 10th day after the third immunization, blood was collected from the orbit to isolate the serum, and an indirect ELISA method was established using the square matrix method to detect serum antibody titers. The optimal antigen coating amount for indirect ELISA was determined to be 0.1 μg. The optimal dilution ratio of negative/positive serum was 1∶1600. Fusion was considered successful when the serum dilution ratio of a positive result was greater than 1:10,000. Three days before fusion, 100 μg of recombinant PRRSV N protein was injected into the abdominal cavity of mice to enhance immunity. After three cycles of immunization, the mouse samples reached 1:25,600, meeting the fusion requirements. Three days after the final immunization, the mice were euthanized, and their spleens were collected under sterile conditions. Spleen and SP2/0 cells were fused at the ratio of 1:6 using PEG 1450. After fusion, the cells were added to the HAT-containing selective medium and cultured in a 37 °C CO_2_ incubator for 1–2 weeks. When fused cells were present, the HT medium was replaced to continue the culture. The preliminarily screened cell lines were subcloned four to six times using the limited dilution method, and the N recombinant protein was used as the antigen. The cell culture supernatant from the wells containing hybridoma colonies was initially screened using ELISA. Following the selection of hybridoma cells, an expanded culture was conducted, resulting in a stable secretion of anti-PRRSV N protein antibodies and a homogenous single-cell population. After cell fusion, two hybridoma cell lines stably secreting monoclonal antibodies (mAbs) against PRRSV N protein were screened. Mice were immunized using hybridoma cells to prepare ascites. Unimmunized BALB/c mice were injected with 0.5 mL of liquid paraffin into the abdominal cavity 3 days in advance to expand the cultured hybridoma cells (the number of cells was approximately 4 × 10^6^), and a 0.3-mL suspension was used to immunize mice intraperitoneally to prepare ascites.

To obtain the N mAb ascites supernatant, the ascites was centrifuged for 15 min at 4 °C at 10,000 × g. An equivalent volume of a 50% saturated ammonium sulfate solution was then added to the supernatant, thoroughly mixed, and incubated for 4 h. Afterward, the sediment was collected. The precipitate was dissolved in phosphate-buffered saline (PBS; 0.01 M, pH 7.4), and the ammonium sulfate was removed via dialysis. The mAb was eluted and collected on a protein G purification column. The antibody titer was determined using the indirect ELISA method established previously and stored at − 20 °C after measuring the concentration.

### Specificity identification of N mAb

After SDS-PAGE electrophoresis of PRRSV N, PRRSV M, FMD/O-VP1, and SVA VP1, the above antigens were transferred to the NC membrane. N mAb was used as the primary antibody, incubated at 37 °C for 45 min, and washed five times with PBST (PBS + Tween-20). The secondary antibody was goat anti-mouse HRP IgG, incubated at 37 °C for 30 min, washed five times with PBST, and colored with enhanced chemiluminescence (ECL).

### N protein labeling with QDFM

The carboxyl-functionalized water-soluble quantum dots were coupled with N protein using the activated ester method. The molar ratios of QDFM to N used for coupling optimization were 1:10, 1:20, 1:30, 1:40, and 1:50. First, 25 μL of QDFM and 6 μL of carbodiimide (EDC) solution (10 mg/mL) were added to a centrifuge tube. Subsequently, 100, 200, 300, 400, or 500 μL of 1.0 mg/mL N protein was added and mixed evenly. The centrifuge tube was fixed on the shaking table and allowed to react for 3 h in the dark at 180 rpm and 25 °C. The product solution was centrifuged for 3 min at 4 °C and 10,000 × g to remove the aggregates of the quantum dots. The QDFM-N coupling compound was obtained by reacting QDFM with N. The final QDFM conjugate was resuspended in 0.5 mL of a 20 mM boric acid solution (BA, pH 8.0) containing 0.1% bovine serum albumin (BSA), 1% sucrose, and 0.1% Triton X-100. The fluorescence intensity of the coupling compound was measured at excitation and emission wavelengths of 302 nm or 585 nm. The solution was kept in the dark at 4 °C until use.

### QDFM test strip assembly

**Fig. 1:**
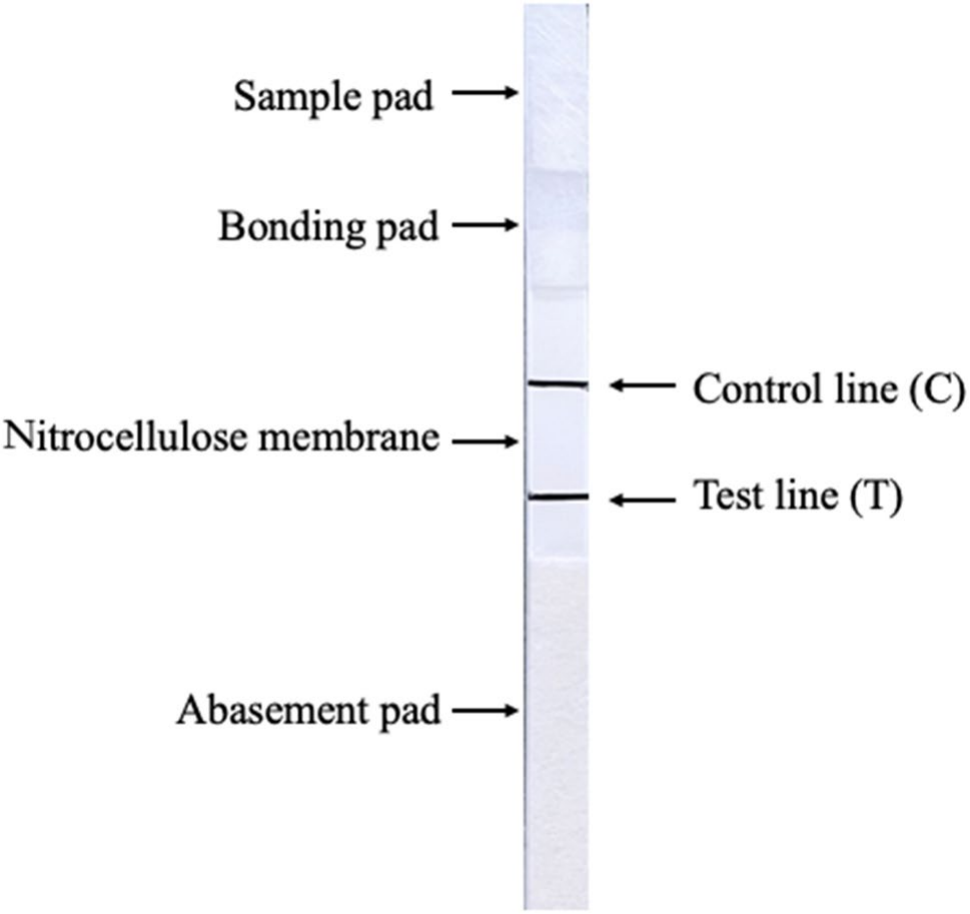
Schematic diagram of the QDFM based multiplex ICS

The QDFM test strip comprised four components, a sample pad, conjugate pad, nitrocellulose membrane, and absorbent pad. After being soaked in 20 mM BA buffer (pH 8.0) containing 0.1% PVP, 0.1% Tween-20, 1% PEG 20000, and 1% BSA, sample pads were dried at 37 °C for 1 h and kept in paper bags with desiccant. Subsequently, purified M protein and N mAb were diluted to 1.5, 1, or 0.75 mg/mL and 1, 0.75, or 0.5 mg/mL, respectively, with 20 mmol/L BA buffer (pH 8.0, containing 1% sucrose), and sprayed on the NC membrane with XYZ3050 three-position spray system at 30 mm/s as the detection line (T line) and quality control line (C line). NC film, sample pad, binding pad, and absorbent paper were pasted on to the PVC fluorescent substrate. Finally, the assembled strip was split into 3.5-mm pieces using a Biodot strip cutting machine (Fig. 1), put in an aluminum foil bag containing desiccant for sealing, and stored in the dark at room temperature (18–25 °C). Using test strips with various coating concentrations, PRRSV standard serum samples were examined to determine the optimal NC membrane coating concentration.

### QDFM ICS reaction principle

**Fig. 2:**
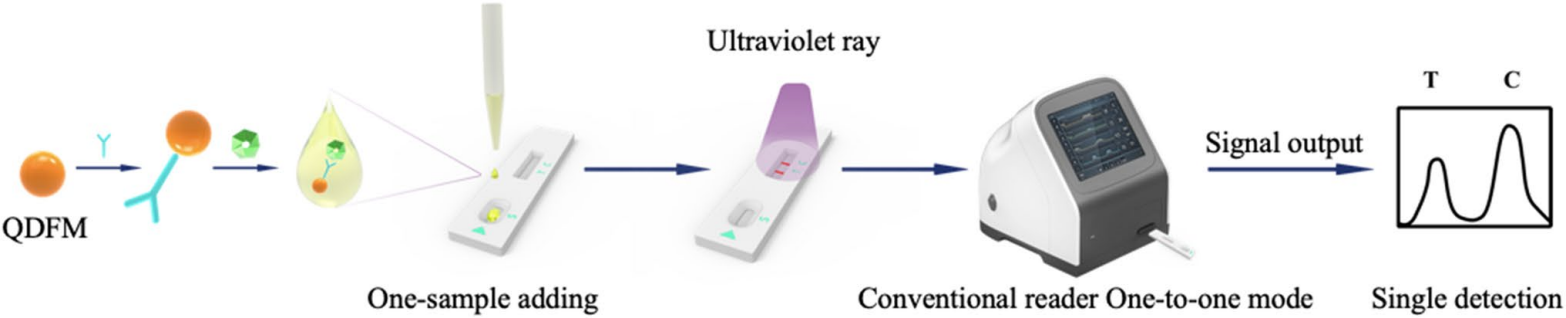
Scheme of conventional one-to-one mode reading lateral flow assay based on QDFM ICS

During testing, serum migration on the sample pad occurs via capillary action. If the serum contains M and N antibodies, an N-Ab-M immune complex forms upon reaching the conjugate pad, where the antibody in the serum sample binds to the N protein and is captured by M on the detection line. A visible orange, fluorescent band will form on the T line as the serum continues to migrate there. Even if the serum includes M or N antibodies, it will still move toward the C line and interact with anti-N mAb complexes to create an orange, fluorescent band visible to the naked eye (Fig. 2).

To test QDFM ICS, serum was diluted with a sample diluent at a ratio of 1:20. Then, 100 μL of the sample solution was added and dropped onto the sample pad of the test strip. The results were then observed using a flashlight containing an excitation wavelength of 365 nm within 15 min at room temperature (18–25 °C). If the T line and C line showed orange bands, it was determined as positive. If only the C line shows an orange band with no bands on the T line, it was determined as negative. If there was no band on the C line, the test strip was judged invalid regardless of whether there was a band on the T line. Alternatively, a fluorescence readout was used to perform qualitative and quantitative analysis (T/C signal-to-noise ratio) on the sample.

### Threshold and detection limit determination of QDFM ICS

To establish the threshold, serum samples from 50 healthy pigs were utilized as negative controls. The test strip results were examined using a portable 365 nm UV light. The T line signal to C line signal ratio (HT/HC) was captured using a fluorescence reader. The HT/HC threshold of the ICS was calculated using the formula: threshold = average ± 3 × standard deviation. The PRRS standard positive serum was diluted with the sample diluent from 64 to 0.125 ng/mL. QDFM ICS was used to detect and determine the extreme value of the test strip using the fluorescence readout.

### Evaluation of QDFM ICS performance

Test strip specificity, sensitivity, and stability were compared to those of a commercial IDEXX-ELISA kit. First, PRRSV-, ASFV-, FMDV-O-, SVA-, PP-, and PR-positive sera, as well as healthy pig serum, were used to test QDFM ICS specificity. Second, serial two-fold dilutions (1:4–1:8,192) of the positive control were used to determine QDFM ICS sensitivity, and a fluorescence reader was used to record the results. Third, to test stability at 0, 1, 2, 3, 4, 5, 6, 7, and 8 months, QDFM ICSs were stored at room temperature (18–25 °C) and 4 °C.

To evaluate the actual effect and accuracy of QDFM ICS, we used the same batch of test strips to test the 189 samples collected, used the commercial ELISA kit (IDEXX) for a comparative test, and compared the results of the two to check the accuracy of QDFM ICS.

## Results

### Production and identification of M and N proteins

**Fig. 3:**
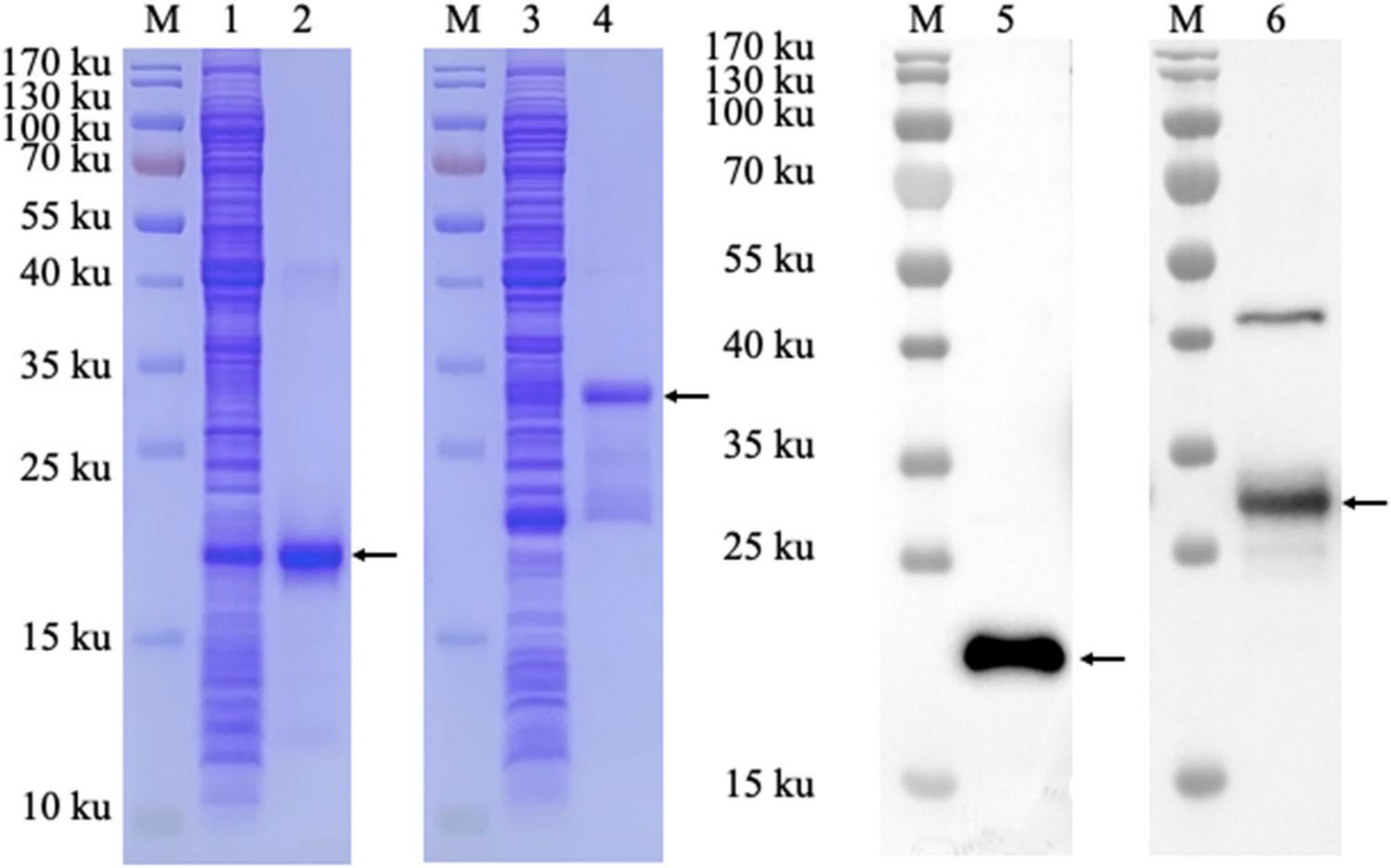
Identification and production of M truncated protein and N recombinant protein. 1, SDS-PAGE analysis of unpurified N recombinant protein; 2, SDS-PAGE analysis of purified N recombinant protein; 3, SDS-PAGE analysis of unpurified M truncated protein; 4, SDS-PAGE analysis of purified M truncated protein; 5, Western- blot analysis of N recombinant protein; 6, Western-blot analysis of M truncated protein, M, Marker

SDS-PAGE and western blotting results indicate that the recombinant N protein and shortened M protein exhibited good reactivity and specificity, with sizes of approximately 19 and 30 kDa, respectively (Fig. 3).

### Purification and specifically identification of N mAb

The collected eluates were subjected to electrophoresis analysis after purification. The SDS-PAGE results demonstrated that the heavy and light chains of N mAb were 50 and 25 kDa, respectively (Figure S2A), indicating that the purified antibody band was correct and high purity. Western blotting revealed that N mAb (1:10,000 dilution) specifically recognized the PRRSV N protein (Figure S2B), indicating a high antibody level was high and specific antigen-binding.

### Titer detection of N mAb

When *OD*_450_ > 0.3, the indirect ELISA results were considered positive. The N mAb titer was 10^4^ (Figure S3), indicating high levels of the antibody and string binding to the antigen.

### Optimization of coating concentration for the NC membrane

**Fig. 4:**
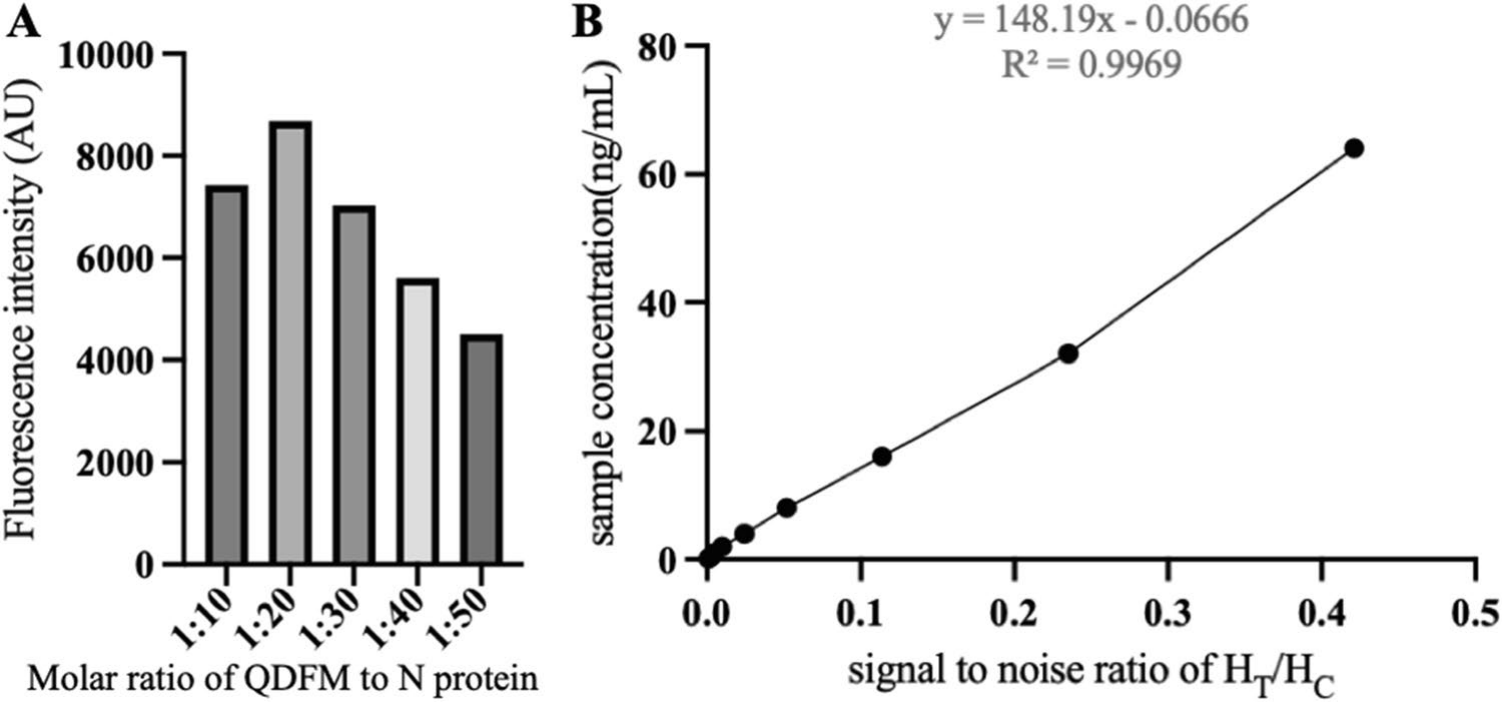
Optimization of the coating concentration for the NC membrane. **A** Fluorescence intensity of QDFM with different molar ratio of N. **B** The correlation between HT/HC and sample concentration

In the process of QDFM-N preparation, the different molar ratios between QDFM and N directly affected the fluorescence intensity of QDFM-N. When the molar ratio of QDFM/N was 1:20, the fluorescence intensity of the coupling was the highest, and the binding was optimal (Fig. 4A).

The optimal coating concentrations were found to be 1.0 and 0.5 mg/L for the test and control lines, respectively. A strong correlation was identified between HT/HC and the sample concentration (Fig. 4B), indicating the feasibility of using QDFM ICS for the detection of PRRSV.

### Specificity and threshold

**Fig. 5:**
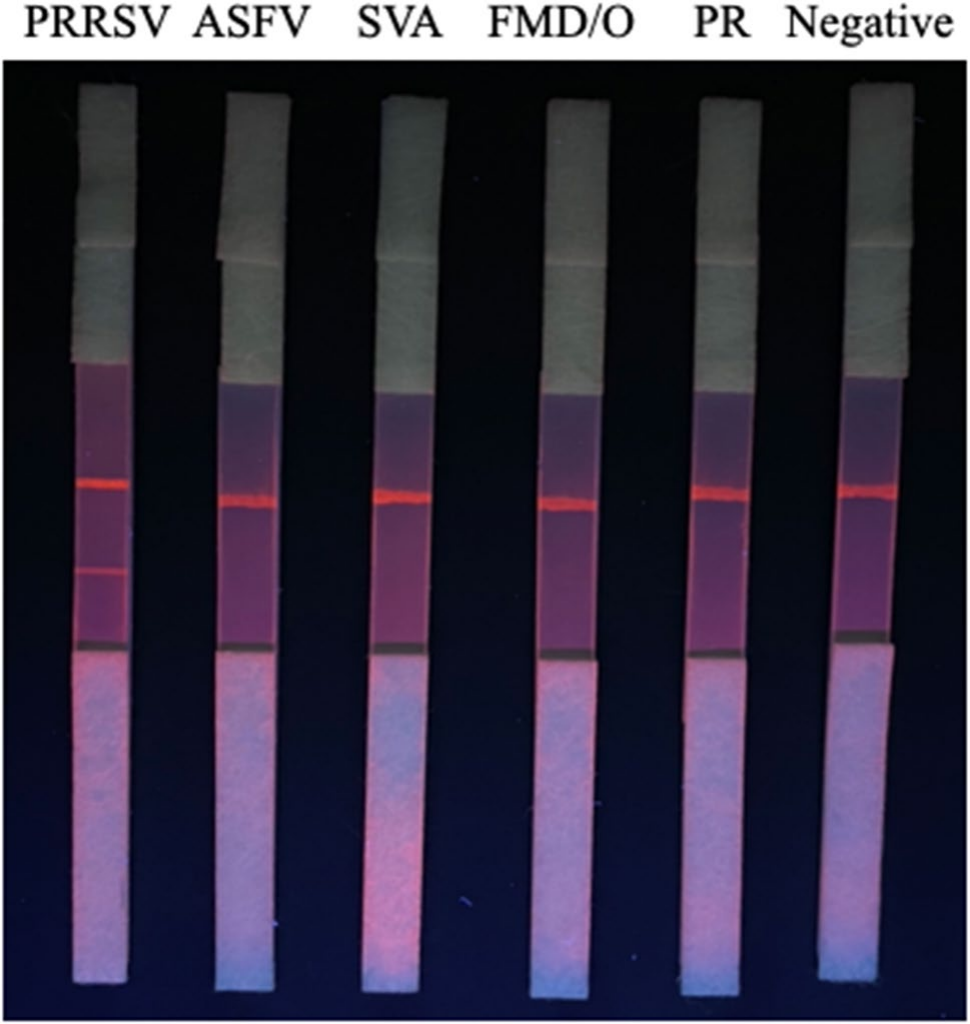
Specificity of the QDFM ICS for PRRSV

A total of 50 healthy serum samples were tested using QDFM ICS, and the results suggested a QDFM ICS threshold of 0.0085. The assay for QDFM ICS was considered negative when the HT/HC value was less than 0.0085 (Table 1). QDFM ICS was used to test PRRSV-, ASFV-, FMDV-O-, SVA-, PP-, PR-positive sera, and healthy pig serum. The results indicated that only serum positivity for PRRSV was detected, with PRRSV samples displaying HT/HC values greater than the threshold. There was no cross-reactivity with other sera (Fig. 5, Table S1).

**Table 1:** The threshold assay of QDFM ICS with 50 healthy PRRSV negative serum samples from pig

Samples	HT/HC				
Negative serum samples	0.0026	0.0044	0.0044	0.0061	0.0028
	0.0051	0.0024	0.0048	0.0028	0.0031
	0.0065	0.0054	0.0034	0.0022	0.0027
	0.0034	0.0041	0.0047	0.0031	0.0057
	0.0050	0.0070	0.0034	0.0053	0.0065
	0.0046	0.0054	0.0030	0.0049	0.0009
	0.0056	0.0054	0.0061	0.0040	0.0057
	0.0034	0.0024	0.0042	0.0037	0.0066
	0.0033	0.0034	0.0062	0.0033	0.0044
	0.0026	0.0045	0.0050	0.0042	0.0051
	Mean = 0.0043				
	Standard deviation = 0.0014				
	Threshold = 0.0085

### Sensitivity and limit of detection

**Fig. 6:**
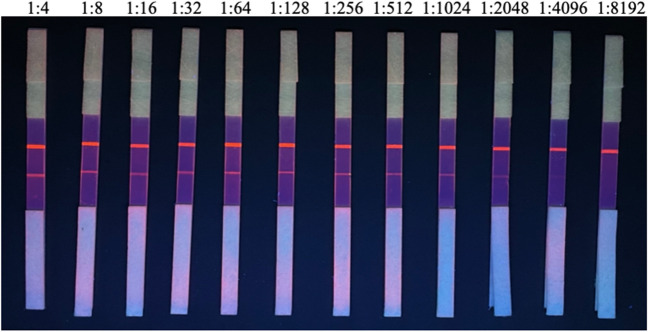
The extreme detection limit of QDFM ICS for PRRSV standard positive serum

PRRSV-positive sera yielded positive results when tested using QDFM ICS at a dilution rate of 1:2,048, indicating that the minimal detectable concentration was 1:4096 (Fig. 6). At a dilution rate of 1:2048, the limit of detection was 1.2 ng/mL, and fluorescence intensity was decreased. This detection limit is comparable to that of commercial ELISA kits. At a dilution rate of 1:4096, the fluorescent line disappeared. These values were accurately measured using a fluorescence reader (Table S2).

### Stability

The test strips were sealed in an aluminum foil bag containing desiccant, and a stability test was performed for both groups of strips over a period of 0–8 months. The results indicated that strips could be stored for up to 6 and 4 months at 4 °C and room temperature (18–25 °C), respectively (Table 2).

**Table 2:** Test strip stability

Months	4 °C (HT/HC)	18–25 °C (HT/HC)	Result
0	0.4725	0.4730	+ , +
1	0.3432	0.2389	+ , +
2	0.2321	0.1217	+ , +
4	0.1079	0.0366	+ , +
5	0.0567	0.0043	+ , −
6	0.0113	0.0035	+ , −
7	0.0040	0.0024	− , −
8	0.0033	0.0026	− , −

### Practicability

A total of 189 clinical serum samples (1:20 dilution) were tested simultaneously using IDEXX commercial ELISA kits and QDFM ICS, with the IDEXX kits considered the standard in all comparisons. Among the 92 samples identified as positive under IDEXX ELISA, 89 were also positive under QDFM ICS, yielding a relative sensitivity of 96.7%. The relative specificity of QDFM ICS was 97.9%. The agreement between QDFM ICS and IDEXX ELISA [(89 + 95)/189] was 97.4% (Table 3).

**Table 3:** Clinical sample detection with QDFM ICS and ELISA

	IDEXX ELISA	
	Positive	Negative
QDFM ICS		
Positive	89	2
Negative	3	95

## Discussion

PRRS is among the most common diseases posing a threat to the global swine industry. PRRSV causes severe infertility, early farrowing, and high rates of late abortion in sows, as well as respiratory illness and mortality in young pigs (Kimman et al. 2009). Despite vaccination efforts, the outcomes have been suboptimal, mainly because most commercially available PRRSV vaccines cannot provide complete protection against genetically diverse strains prevalent on farms. Moreover, PRRSV exhibits continual evolution to evade existing immunity in vaccinated herds, causing new outbreaks (Lunney et al. 2016; Mengeling et al. 1998; Tian et al. 2007). Therefore, developing a rapid diagnostic procedure is essential for disease prevention and treatment.

Serological testing is performed to confirm viral infection and for epidemiological surveillance. Commonly used serological techniques for antibody detection include ELISA, IFA, western blotting, and colloidal gold immunochromatographic detection. Although ELISA and colloidal gold ICS are often used in laboratories (Kashyap et al. 2020; Hu et al. 2021), both methods present limitations. In pigs who have recovered from the infection, ELISA is a reliable approach for identifying PRRSV-specific antibodies (Kashyap et al. 2020); however, it can be costly, time-consuming, and requires skilled professionals and specialized equipment. Colloidal gold ICS offers the advantages of simplicity, rapidity, equipment-free operation, and affordability; however, its sensitivity is relatively low (Li et al. 2019). Compared with colloidal gold or other labeling techniques, QDFM conjugates, characterized by fluorescent microspheres, offer various advantages including good photochemical stability, multiple and long-term light source excitation, strong fluorescence, and resistance to quenching (Kong et al. 2021; Yang et al. 2011). Notably, the results are visible in 5–10 min to the naked eye. Furthermore, an ICS based on QDFM could be used for qualitative analysis in the field and quantitative analysis in the laboratory with specific instruments. Hence, this technique is suitable for rapid high-throughput detection in rural areas or on-site, applications and is significant for serological diagnosis.

Choosing appropriate target antigens is critical for developing a diagnostic assay. The M protein, a main structural protein of PRRSV, contains multiple antigenic epitopes, inducing robust neutralizing antibodies (Wang et al. 2014; Yang et al. 2000). Hu et al. (2021) utilized the truncated M gene expressed in a plasmid vector and colloidal gold immunochromatography for detecting PRRSV antibodies. In the early stages of viral infection, the M protein is recognized by specific antibodies that remain stable for an extended period (Hicks et al. 2018), suggesting that M protein levels serve as a potential biomarker for early PRRS diagnosis. In addition, the N protein of PRRSV, possessing the highest immunogenicity, represents another suitable target. The N protein is crucial to the viral life cycle because it controls host cytokine levels while interacting with other viral proteins and RNA (Cai et al. 2009; Dea et al. 1996). Yu et al. (2018) applied colloidal gold nanoparticle–labeled dual-type nucleocapsid proteins encoded by N protein for PRRSV diagnosis. In our study, soluble recombinant M and N proteins were induced in the prokaryotic expression system. The purification of soluble recombinant M and N proteins ensured their immunogenicity, and N mAb was prepared. N mAb can specifically recognize the PRRSV N protein and avoid non-specific reactions in cell lines (Kong et al. 2021; Li et al. 2019). Based on this, we developed a QDFM test strip that can rapidly detect serum PRRSV antibodies. The PRRSV N protein was coupled with QDFMs as a tracer, while anti-N mAb and M protein served as the quality control and detection lines, respectively.

In the present study, specificity evaluation of the QDFM ICS reaction revealed visible bands only for PRRSV at the T line, while other viruses were not detected. Notably, the QDFM ICS exhibited higher sensitivity than other immunochromatographic strips for field tests. The minimum detection limit was 1.2 ng/mL when the maximum dilution rate was 1:2048, comparable to that of ELISA kits. The colloidal gold ICS developed by Zhou et al. (2009) showed a similar detection limit when serum samples were diluted to 1:640. Our QDFM ICS assay, with a detection limit at a 1:4096 dilution, exhibited an analytical sensitivity sixfold higher than that of the colloidal gold ICS. Moreover, at a 1:5 sample dilution, the colloidal gold ICS developed by Yu et al. (2018) demonstrated similar sensitivity (97.5% vs. 97.3%) as the commercial IDEXX ELISA. Our QDFM ICS is comparable to ELISA kits under lower sample dilution conditions, and its detection sensitivity is comparable to that described in other reports on QDFM ICSs (Kong et al. 2021; Li et al. 2022a, b). In addition, unlike the gold-colloidal method, the quantum dot is unaffected by hemoglobin color, and QDFM increases the sensitivity and enlargement of the light signal of antigen–antibody–specific binding compared to other labeling methods (Kong et al. 2021; Yang et al. 2011). Notably, further validation of the performance of our QDFM ICS assay with 189 serum samples (1:20 dilution) indicated that the sensitivity, specificity, and accuracy of the assay compared to those of PRRSV ELISA were 96.7%, 97.9%, and 97.4%, respectively. In summary, our QDFM ICS assay is ideal for non-laboratory point-of-care PRRSV detection, an especially relevant issue for pig herds in rural locations.

In conclusion, the QDFM ICS developed in this study is suitable for on-farm use, with strip-test results generated successfully in a short period using trace swine serum. QDFM ICS accurately detected PRRSV antibodies, and no cross-reactivity was observed with other sera. Based on 189 clinical samples, QDFM ICS demonstrated equivalent sensitivity, specificity, and accuracy to a commercial ELISA kit, demonstrating its effectiveness for detecting PRRSV in clinical samples. Moreover, QDFM ICS is simple to operate and does not require complicated sample processing. Thus, it is promising for clinical applications in low-income regions. In conclusion, this detection technology has important diagnostic implications for serology.

## Supplementary Information

Below is the link to the electronic supplementary material.

Supplementary file1 (PDF 297 KB)

## Data Availability

The authors declare that the data supporting the findings of this study are available within the article.
