# Supplementary material for: Quantum dot fluorescent microsphere-based immunochromatographic strip for detecting PRRSV antibodies
Source: Appl Microbiol Biotechnol. 2024 Apr 4;108(1):283. doi: 10.1007/s00253-024-13125-2 (PMC10995003; doi:10.1007/s00253-024-13125-2)
Supplement: Supplementary file 1 — Supplementary file1 (PDF 297 KB) [file 253_2024_13125_MOESM1_ESM.pdf]

Journal name:

Applied Microbiology and Biotechnology

Manuscript Title:

Quantum dot fluorescent microsphere-based immunochromatographic strip for detecting PRRSV antibodies

The name(s) of the author(s):

Rui Yang[1,2,3] Yi Ru[2] Huibao Wang[4] Rongzeng Hao[2] Yajun Li[2] Tao Zhang[4] Haixue Zheng[2] Yong Zhang[1] Xingxu Zhao[1]

The affiliation(s) and address(es) of the author(s):

[1] College of Veterinary Medicine, Gansu Agricultural University, Lanzhou 730070, Gansu, China

[2] State Key Laboratory for Animal Disease Control and Prevention, College of Veterinary Medicine, Lanzhou University, Lanzhou Veterinary Research Institute, Chinese Academy of Agricultural Sciences, Lanzhou 730000, Gansu, China

[3] China Agricultural Veterinarian Biology Science and Technology Co. Ltd., Lanzhou 730046, Gansu, China

[4] College of modern Agricultural Engineering, Gansu Forestry Technological College, Tianshui 741020, Gansu, China

The e-mail address, telephone of the corresponding author:

Rui Yang: yr051049@163.com, Tel: 15095439827

Yong Zhang: zhychy@163.com, Tel:13893126652

Xingxu Zhao: zhaoxx0931@163.com, Tel:13609328702

M (ORF6: 309 bp)
GGGTCGTCTCTAGACGACTTTTGCCATGATAGCACGGCTGGTGGTGGTGGT
TCTGGTGGTGGTGGTTCTACCTCCAGATGCCGTTTGTGCTTGCTAGGCCGC
AAGTACATTCTGGCCCCTGCCCACCACGTCGAAAGTGCCGCGGGCTTTCAT
CCGATTGCGGCAAATGATAACCACGCATTTGTCGTCCGGCGTCCCGGCTCC
ACTACGGTTAACGGCACATTGGTGCCCGGGTTGAAAGGCCTCGTGTTGGGT
GGCAGAAAAGCTGTTAAACAGGGAGTGGTAAACCTTGTCAAATATGCCAA
ATAA
N (ORF7: 372 bp)
ATGCCAAATAACAACGGCAGGCAGCAAAAGAAAAAGAAGGGGAATGGCC
AGCCAGTCAATCAGCTGTGCCAAATGCTGGGTAAGATCATCGCCCAACAAA
ACCAGTCCAGAGGCAAGGGACCGGGGAAGAAAAATAGGAAGAAAAACCC
GGGGAAGCCCCATTTCCCTCTAGCGACTGAAGATGACGTCAGGCATCACTT
TACCCCTAGTGAGCGGCAATTGTGTCTGTCGTCGATCCAGACTGCCTTCAAT
CAGGGTGCTGGAACTTGTGCCCTGTCAGATTCAGGGAGGATAAGTTACACT
GTGGAGTTTAGTTTGCCGACGCAACATACTGTGCGTCTGATCCGCGCCACA
GCGTCACCCTCAGCATGA

Figure S1 Analysis of nucleotide sequence of M truncated protein and N recombinant protein

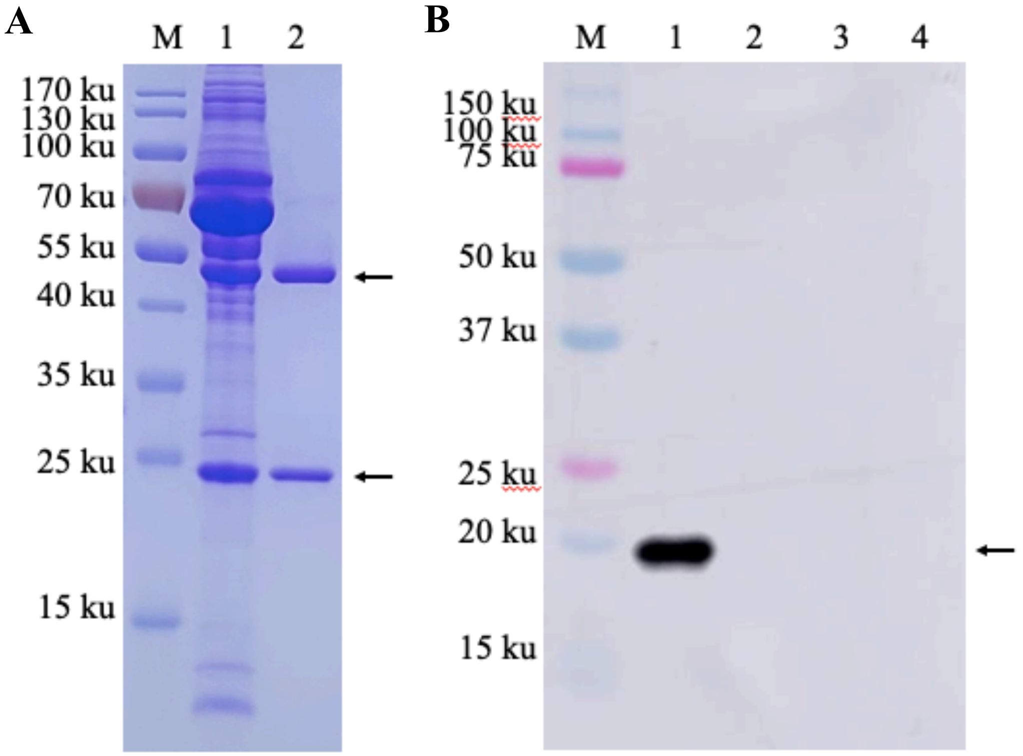

Figure S2 Identification and production of N mAb. A: 1, SDS-PAGE analysis of unpurified N mAb, 2, SDS-PAGE analysis of purified N mAb, M: Marker (26616); B: 1, Western-blot analysis of PRRSV N, 2, Western-blot analysis of PRRSV M, 3, Western-blot analysis of FMD/O-VP1, 4, Western-blot analysis of SVA-VP2, M: Marker(1610374).

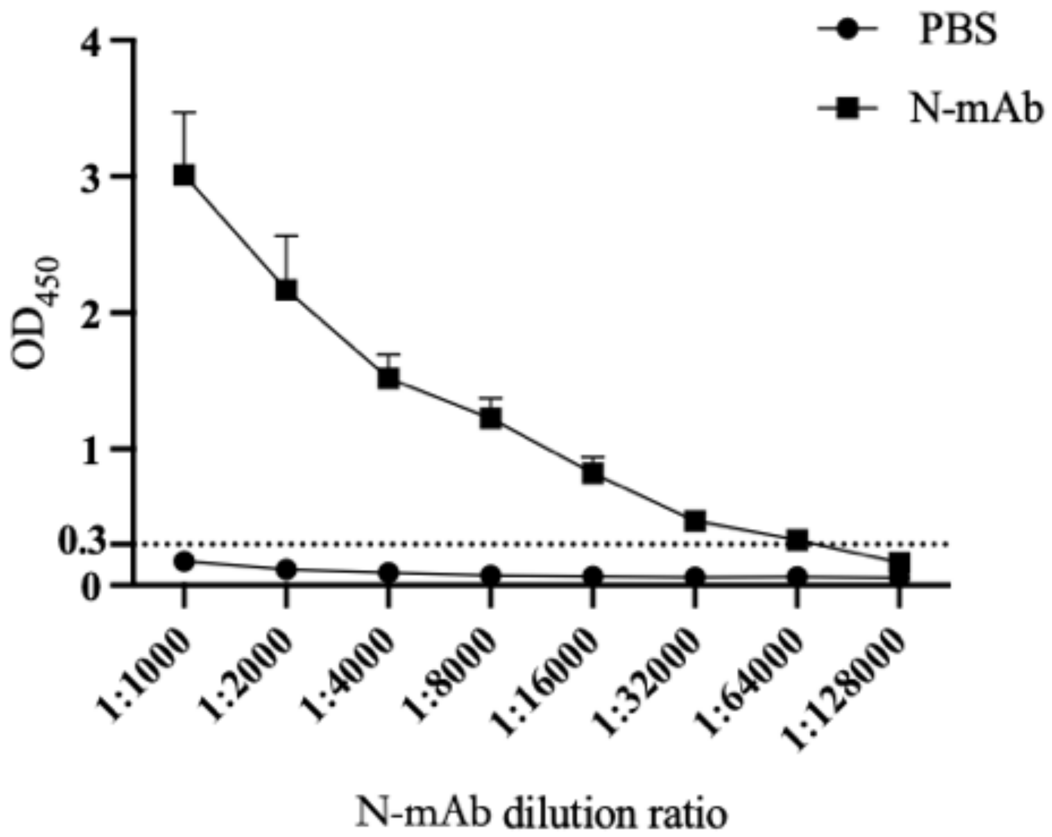

Figure S3 Titer detection of N-mAb

Table S1 Specificity of the QDFM ICS for PRRSV

Samples	HT	HC	HT/HC	Result
PRRSV	8342	13938	0.5985	+
ASFV	43	14996	0.0029	-
FMDV-O	38	14835	0.0046	-
SVA	55	14673	0.0037	-
PR	40	14789	0.0047	-
Negative	51	13018	0.0039	-

Table S2 Sensitivity assay of QDFM ICS testing of PRRSV

Dilution	HT	HC	HT/HC	Result
1:4	8732	14387	0.6069	+
1:8	7394	14218	0.5200	+
1:16	6458	14187	0.4552	+
1:32	6239	14223	0.4387	+
1:64	5648	14322	0.3944	+
1:128	4415	14087	0.3134	+
1:256	3129	14111	0.2217	+
1:512	2725	14215	0.1917	+
1:1024	1575	14337	0.1099	+
1:2048	697	14105	0.0494	+
1:4098	64	13979	0.0046	-
1:8196	45	14036	0.0032	-
